# Increasing Reasoning Awareness: Video Analysis of Students’ Two-Party Virtual Patient Interactions

**DOI:** 10.2196/mededu.9137

**Published:** 2018-02-27

**Authors:** Samuel Edelbring, Ioannis Parodis, Ingrid E Lundberg

**Affiliations:** ^1^ Division of Community Medicine Department of Medical and Health Sciences Linköping University Linköping Sweden; ^2^ Department of Learning, Informatics, Management and Ethics Karolinska Institutet Stockholm Sweden; ^3^ Rheumatology Unit Department of Medicine, Solna Karolinska Institutet, Karolinska University Hospital Stockholm Sweden

**Keywords:** medical education, clinical decision making, problem solving, computer-assisted instruction

## Abstract

**Background:**

Collaborative reasoning occurs in clinical practice but is rarely developed during education. The computerized virtual patient (VP) cases allow for a stepwise exploration of cases and thus stimulate active learning. Peer settings during VP sessions are believed to have benefits in terms of reasoning but have received scant attention in the literature.

**Objective:**

The objective of this study was to thoroughly investigate interactions during medical students’ clinical reasoning in two-party VP settings.

**Methods:**

An in-depth exploration of students’ interactions in dyad settings of VP sessions was performed. For this purpose, two prerecorded VP sessions lasting 1 hour each were observed, transcribed in full, and analyzed. The transcriptions were analyzed using thematic analysis, and short clips from the videos were selected for subsequent analysis in relation to clinical reasoning and clinical aspects.

**Results:**

Four categories of interactions were identified: (1) *task-related dialogue,* in which students negotiated a shared understanding of the task and strategies for information gathering; (2) *case-related insights and perspectives* were gained, and the students consolidated and applied preexisting biomedical knowledge into a clinical setting; (3) *clinical reasoning interactions* were made explicit. In these, hypotheses were followed up and clinical examples were used. The researchers observed interactions not only between students and the VP but also (4) *interactions with other resources,* such as textbooks. The interactions are discussed in relation to theories of clinical reasoning and peer learning.

**Conclusions:**

The dyad VP setting is conducive to activities that promote analytic clinical reasoning. In this setting, components such as peer interaction, access to different resources, and reduced time constraints provided a productive situation in which the students pursued different lines of reasoning.

## Introduction

In professional education, students need to apply facts and concepts into relevant work-life situations. For medical students, it can be challenging to apply biomedical knowledge when entering into clinical practice; this application has previously been described as “slow, awkward, or absent” [1]. It is therefore important that students are assigned activities that guide the transition from comprehension to higher-level problem solving and management [2]. Educational researchers suggest that reasoning skills can and should be taught in order to develop deeper understanding of facts and concepts [2]. In the context of medicine, clinical experiences and thorough biomedical knowledge are combined within clinical reasoning, thus facilitating diagnostic and management processes in relation to patients [3]. The nature of clinical reasoning has been thoroughly researched; yet, in our experience, it is still rare for medical educators to arrange learning activities that enable any insight into, or guidance of, students’ reasoning processes.

Two models are commonly used to describe the nature of clinical reasoning processes. The hypothetico-deductive model describes reasoning as starting with the generation of hypotheses, followed by analytic evaluation of these hypotheses [4]. This model is firmly rooted in laboratory and experimental empirical settings. However, research based on more naturalistic, real-life professional situations has challenged the hypothetico-deductive model and proposed more intuitive and experience-based nonanalytic models, often termed pattern recognition models [5]. In clinical professional practice, the nonanalytic pattern recognition model is emphasized because of the multidimensional characteristics of real-life practice [3]. This type of reasoning requires experience from clinical examples that generates an array of analogies as students develop their expertise [6]. However, in undergraduate education, students do not have large repertoires of patient encounters and need to rely on analytic use of their biomedical knowledge. The two reasoning approaches are not mutually exclusive; either one can be used or both in tandem, depending on the context and the educational goal [7,8].

An interactive virtual patient (VP) allows students to gather information in a stepwise manner and suggest diagnosis and management. Relevant VP cases have been shown to engage students in active thinking and decision making [9-11]. The engagement and perceived relevance are important to support meaningful learning [12]. VP activities are often designed with flexible, individual self-study in mind. However, one could assume that peer settings, in which students need to verbalize and argue their standpoints, would make reasoning processes more discernable to students and thus support their learning of reasoning strategies. In complex clinical settings, decisions are often based on collaborative reasoning [13]. Collaborative-thinking processes have been emphasized in complex processes such as managing a large military vessel [14], and the philosopher Dewey considered dialogue fundamental to logical thought [15]. Collaborative reasoning is therefore both a means to gaining professional competence and an educational goal for students in terms of gaining awareness of their own critical thinking [16].

The use of computer applications in small group settings has generally been shown to be beneficial for learning [17]. The dyad, which is a two-party peer collaboration setting, has been shown to generate learning outcomes at more abstract levels in problem solving than if the same task had been performed individually [18]. The dyad reasoning setting may also grant educators access to reasoning processes, thereby making it possible to refine and design engaging and challenging situations. Increasingly complex patient scenarios and challenges in health care have intensified the need for shared reasoning and collaboration in professional practice [13,19]. The aim of this study was to explore characteristics of medical students’ two-party reasoning on clinical cases presented as computerized VPs.

## Methods

An exploratory observation was conducted to identify interactions and delineate their characteristics during VP case sessions performed by students in dyads. The students were third-year medical students during their clinical rotation at the Rheumatology Unit at the Karolinska University Hospital in 2011. Four VP cases constituted a mandatory task, which was recommended to be conducted in pairs. The VP assignment was not scheduled at a specific time or graded, but it served as a basis for discussion with a clinical supervisor at the end of the rotation. The VPs were based on authentic patients and authored in a derivative platform of the NUDOV system described in Wahlgren et al [20]. The main researcher (SE) recruited a convenience sample of 2 student pairs (all female) and obtained written informed consent to video-record their VP sessions. They were free to select one of the 4 VPs. Two different cases were selected, one by each of the two pairs. Each session lasted for approximately 1 hour.

The construction of themes was data-driven, that is, not directed by a priori categories. The first session (session #1) was transcribed in full and a preliminary thematic analysis was performed [21]. This analysis was initiated by the first author, followed by iterative analysis in collaboration with the coauthors. In the first phase, instances of interaction pertaining to learning and clinical reasoning were identified as themes. These themes were then used to identify corresponding instances in the second session (session #2). Emerging themes along with illustrative instances from the videos were analyzed collaboratively in two collaborative data analysis sessions [22]. The research group brought specialty-specific (rheumatology) and educational perspectives into the analysis based on their expertise. Different views and perspectives on the themes were resolved by consensus. Clinical information of the VPs is provided in Multimedia Appendix 1.

Ethical approval was granted by the regional Ethics Review Board in Stockholm, Sweden (#2009/609-32/5).

## Results

### Overview

Four categories of interactions related to learning were identified: (1) task-related dialogue, (2) case-related insights and perspectives, (3) clinical reasoning interactions, and (4) interactions with other resources. Each category is presented below, and interactions from the different categories are illustrated using quotes from the two sessions, indicated by the session number and point in time of the respective session.

### Task-Related Dialogue

Part of the dialogue was dedicated to understanding how to approach the task and navigate in the software. Interactions in this category were related to, for example, the students’ perceptions on how the assignment would be followed up by their supervisors, and, more directly, how the interface worked, in particular where they had to click to navigate in the VP software:

Yes, exactly. May I just ask: what are we going to report on Friday? It was going to be about the diagnosis we identified and then about the management, right?

Exactly.

OK, I just wanted to be sure.

#1, 09:25

But, by the way, did we have any...It says 3 questions, but we could ask many questions, couldn’t we?

Yes, it...

Whether he is on any medication, perhaps?

Yes, but the question is...do you think it will register it?

No, but if we just imagined.

Yes, we can make up questions.

#2, 05:50

I was just thinking...should we click through step by step? Or we can just adopt a specific approach.

Yes, perhaps we should just choose what’s relevant and then look at things again later.

But for now we’ll follow these ones, anyway.

Yes...

#1, 17:49

Since only one person could select and write text into the software, there were negotiations about control, for example, what to select and what questions to put to the patient. Sometimes, the pairs divided tasks between themselves. For example, one could read on the screen while the other one looked up facts in a textbook:

It might be far-fetched, but...should I read all blood test results?

Here: it should be below, or between 60 and 400, but go ahead and read. Keep reading.

I’ll read quietly, so that you can check there.

OK.

#1, 56:33

What more do we want to know? For the purposes of our own learning...We want to know more...about how the pain varies?

Yes, and when. Whether he suffers from morning stiffness, whether motion relieves it and whether rest worsens the pain.

I want to know if he is affected in any other way, if he has any other symptoms, any other...

#2, 19:16

### Case-Related Insights and Perspectives

Through interaction with the VP cases, the students obtained insights related to symptoms and diagnoses and identified new clinical perspectives. The process of identifying differential diagnoses and the progress toward the final diagnosis generated discussions and reflections, based on information obtained from the patient and clinical findings. The software and the way the VP cases were constructed allowed a free flow of ideas, several of which were followed up at later stages. Students reflected upon differences between this setting and authentic patient encounters, which they perceived as more constrained because of time restraints. They referred to previous experiences of feeling pressured to appear as if they already had knowledge in front of patients and supervisors:

See how much we are able to think about when sitting like this. When you are with a patient so ehh...

It’s because you don’t have a lot of time. You have to focus on behaving properly in front of the patient and so on.

#2, 33:10

There was plenty of time to elaborate on findings. Ideas and hunches could be followed up. The clinical information in the VP case was presented in a variety of ways namely, in text, short video clips of the patient answering questions, or filmed examination procedures. In session #2, the students were inspired to try out a practice physical examination on themselves while watching the procedure, and they watched the procedure one more time after that:

Well, let’s check him over, okay?

Yes, I agree.

At this point, an examination of the patient’s (Carl) chest flexibility is displayed in the software: “Carl, now I would like you to breathe deeply while looking at me. Please, breathe so that my hands move.”

Then, it should normally move like this, right?

The student shows her hands moving.

May I try it on you?

The student performs the examination on her peer, who is breathing deeply in and out.

Somewhere here?

Yes.

#2, 36:48

Schober’s test.

Film clip showing the examination of the patient’s back flexibility is displayed.

So he is just bending the hip joints, not like that.

Yes, exactly.

Exactly, not like this when he also is bending the back.

The student illustrates different types of back flexion using her hands.

Can we look at it again? I would like to see it one more time.

#2, 39:47

In one case, the reference of nonsteroidal anti-inflammatory drugs (NSAIDs) led to a discussion about the mechanism of action of such drugs and their effects in relation to the assumed diagnosis:

Yes, because it is an NSAID.

Yes, exactly.

But, oh my god, isn’t this weird? Well, NSAIDs dampen the inflammation, but we want to prevent even more, don’t we?

So you want to give corticosteroids, or something like that?

Yes, or wait, was he still on that? The white blood cell count was high as was the sedimentation rate...We don’t want to just stop the symptoms, right? We want to stop the progression, don’t we? If you understand what I mean...

Yes, I understand, but NSAIDs are anti-inflammatory drugs, this is what they do.

Well, yes, but it’s only COX that is inhibited.

Yes, it’s quite a weak inhibition of the inflammation one could say.

Then it’s only leukotrienes and prostaglandins that are not being produced, which means that you don’t see...

Leukotrienes are produced, it’s the prostaglandins [that are not produced], and thromboxanes are not produced either.

Correct, it’s only those that are not produced.

But still, it [the drug] inhibits quite a lot of the inflammation, one could say.

Yes, that’s true.

But, yes, it’s not the same thing as corticosteroids or methotrexate, or things like that.

It does not inhibit the lymphocytes per se, even if not as many of them stream out, maybe.

Yes, you are not supposed to see the same upsurge.

But again, we want to prevent something severe here so that it doesn’t result in a bamboo spine, which is permanent.

Yes, I understand what you mean, but at the same time I think that there must be a reason why they treat it this way. But we could read more about it maybe. It is also stated here that continuous physical exercise prevents worsening of the functional status, so I note this recommendation: exercise!

#2, 1:07:34

The VP cases were based on authentic patients; they were therefore not textbook examples. Students identified inconsistencies in relation to classification criteria; yet, their suggestion of diagnosis at different stages during the session—rheumatoid arthritis (RA) in the following instance—was also based on references from real-life clinical complexity:

I thought of RA.

I also thought of that.

But isn’t it small joints that are affected first?

Yes, I also thought about that, and he mostly seems to have problems with large joints.

But we could note it down as an alternative diagnosis. Not everything has to be according to the textbooks.

Yes, true.

#2, 44:41

The students brought previous knowledge into the reasoning. However, in many instances, the level of knowledge varied between the 2 individuals, and on several occasions it was incomplete. They supported each other by filling knowledge gaps and looked up information when they were uncertain:

This sounds pretty much like Bechterew to me.

I mean, I don’t remember so much about that disease. Could you remind me?

Sacroiliitis, and some other things...

#2, 11:03

I always confuse CRP and ESR, which is which...One is supposed to be below 100, and the other one below 3...I think.

Yes, but the one with 100 – it’s only when you have a bacterial infection that it can be over 100.

That was it. CRP, right?

#1, 54:08

### Clinical Reasoning Interactions

The clinical reasoning interactions consisted of uncertainty, questioning, clarifying, and verifying dialogues. The dyad setting encouraged the students to generate explicit hypotheses, as well as to proceed with confirming or rejecting these hypotheses. In both sessions, there was often one and the same peer giving suggestions to further advance the reasoning:

OK, then I think we can decide that he most probably has PMR, and so I think we initiate him with glucocorticoids to see whether he gets better. Because this way we can confirm the diagnosis, right?

Yes, sure.

#1, 52:57

The following quote provides another example of how the reasoning is verbalized and the thread of the reasoning is made explicit. The students updated themselves on diagnostic criteria, and this information guided both their focus when further interviewing the patient and their interpretation of the patient’s answers and other findings. The reasoning revolved around symmetry and the patient’s ways to express the location of pain.

Should we consider polymyalgia rheumatica?

The student looks up the classification criteria in the textbook.

Yes, I am not sure what...

I will check what it is.

Okay. But here it is stated that chronic idiopathic myositis can be an isolated inflammatory systemic disease, or part of another rheumatic disease such as Sjögren’s syndrome, systemic sclerosis, mixed connective tissue disease, systemic lupus erythematosus or rheumatoid arthritis.

Yes...

And then with regard to polymyositis in particular, it is stated that the predominant symptoms are decreased muscle strength, decreased stamina in the proximal muscles, shoulders, nape of the neck, thighs, and the pelvis. Symmetric distribution. So we need to know whether it is symmetric. Myalgia may occur, but it is not as common as the weakness.

Was there no question about the symmetry?

The student browses through the VP case in the software.

No, not really.

And then it is stated that acute myositis, which mostly is seen in conjunction with viral infections, is established quicker and is often followed by myalgia. So it could be this.

I’m sorry, what did you say?

Acute myositis, which is mostly seen in conjunction with viral infections, is established quicker and is often followed by myalgia. So it could definitely be this, too.

That's true. And this was that...poly- and dermatomyositis?

Yes, exactly.

And he responds quite inadequately to the question about symmetry: “It is located in the shoulders and hips.” So it should be that...Because otherwise, he would say: “the right shoulder.” [The student pats her shoulder] and...

Hm, yes, it should be that.

And here, for polymyalgia rheumatica it is stated that [the student reads from the textbook] “new onset of relatively acute established mechanic pain in the proximal parts of the arms, shoulders, nape of the neck, and/or hip areas and thighs is characteristic of PMR, as it is abbreviated. Patients describe intense morning stiffness, difficulties turning over in bed, getting up out of bed, and putting on clothes during the morning hours.”

Okay.

In general, the symptoms are fully developed within several days to a couple of weeks. Constitutional symptoms, such as fatigue, subfebrility, loss of appetite, and weight loss...

Well...hehe.

It’s crystal clear!

#1, 23:32

Furthermore, the students verbalized interpretations of radiographic images. In session #2, radiographic visualization of the spine evoked interest, and the peers helped each other to understand the findings and relate them to the patient’s symptoms. The software displayed the images, but there were no indications of what to look for (eg, arrows), or how to interpret the findings. The students realized that they could not fully interpret one of the images:

Yes. Here, we can see a little better. Let’s see. Here, it is very uneven; and here, it feels like it starts to become more linear.

Hm, there they are evened out. They are evened out there.

These ones stick out like that...

And they are also evened out, I think.

Yes, exactly.

Here, however, you can see that it’s fine.

#2, 59:00

Next radiographic image

Still evened out, that is what I see.

Yes.

Yes, now we are up there.

Yes, the cervical spine.

This one is very difficult to interpret, I think.

I find it absurdly difficult, too. Yes.

Has it grown together here? And here, maybe? Here too. I don’t think I am competent enough to interpret that one, actually.

No, that’s true. I’m not competent enough for that one either. I mean, I’m not saying that you are not competent; I’m just saying that I’m not competent.

#2, 59:25

The students worked on the VP cases in conjunction with other clinical tasks during their clinical rotation. Several times, they referred to patients and procedures they had seen before. Examples from real-life experiences at the clinic were used to illustrate representative instances in the VP cases and facilitated clarifications during reasoning:

Is it possible to have psoriatic arthritis without the typical skin lesions?

I think it is, but I think it most commonly affects the DIP [distal interphalangeal] joints.

The psoriatic arthritis?

Yes, or am I wrong?

Yes, they are the ones most commonly affected, but it can also be...I mean...the man I saw earlier today, he had...

Yes?

Well, I was not responsible for him on my own, but I talked to him for a while. Both of his wrists were swollen, here, and here, and he had pain in one shoulder, an elbow, and in both of his feet; so it was quite extensive.

Yes. Did he have any other symptoms?

No, those specific joints were swollen and tender.

#2, 47:04

### Interactions With Other Resources

Resources other than the VP platform were also used; mostly a textbook but also Web-based medical resources, lecture notes, and a list of laboratory tests. The students were allowed to use other resources, and they did so when they found it helpful in making the diagnosis, when they wanted to relate content in the VP case to classification criteria, guidelines, and common management routines, and when identifying knowledge gaps:

They were like evened-up corners of the vertebrae, I think.

Yes, I remember that. But what is the source of the pain? I mean, what’s happening? What’s the reason for the patient experiencing pain? Well, I think I should read a little about it in the textbook. I’ll look it up. [The student opens the textbook].

Well, yes, we can have a look; it’s something we should know anyway.

#2, 11:32

This is, in fact, an awesome way to learn!

Yes, definitely.

Especially when you have such a good textbook.

Yes, it’s actually a really good one.

Imagine if we had such a good textbook during all rotations.

It’s actually extremely useful to work in a problem-based manner sometimes.

#1, 1:07:02

Hm, okay. But wait...should we look at...what's that? Is the ESR [erythrocyte sedimentation rate] elevated?

55.

And it is supposed to be below 3, isn’t it?

Yes, normally yes...

Well...we should look it up.

The student uses the Internet.

Yes.

Okay.

I will just check.

Ah...

My goodness, that was very slow...

My son had an ESR of 29"...Okay, it is supposed to be below 8.

#1, 53:25

## Discussion

### Principal Findings

In this study, we identified and described interactions within student dyads in VP-based learning sessions. Our observations revealed elaborate reasoning processes supporting the development of analytic clinical reasoning. Overall, the dyad peer setting contributed to fruitful interactions and promoted the development of analytic expertise.

### Task-Related Dialogue

The assignment that framed the students’ task was loosely regulated by the teachers, and the VP interface allowed for relatively free exploration of the patient cases, which made the task-related dialogue between the students necessary. During the task-related dialogue, a shared understanding was created on how one should approach the task to gather patient data in the case-related context.

### Case-Related Insights and Perspectives

Diagnosis-specific facts were elaborated upon, reference values from previous experience and other resources were used, and a variety of procedures were observed and discussed. The evaluation of key findings had positive consequences and resulted in structured gathering of further information and suggestions for managing the respective patient. To some extent, the knowledge was already present and readily available in the students’ reasoning. However, in several cases, the students had to search for information or ask each other. The verbalization and application of knowledge seemed to add further value to preexisting knowledge, since it was put into a clinical context. Biomedical facts were thus interwoven with the clinical case in a very active manner, connecting knowledge and procedures in a meaningful way.

### Clinical Reasoning Interactions

The verbal interaction between students made it possible to elicit reasoning processes that otherwise might have remained implicit or would not have occurred. Some of the findings pertain to a specific case while others are more general, for example, processes related to the development of reasoning strategies or decision making.

Previous literature on clinical reasoning is generally characterized by categorization into hypothetico-deductive analytic approaches and experiential-based nonanalytic approaches [7]. In our data, a slowed-down analytic reasoning was salient. The students made efforts to elaborate upon clinical findings in relation to classification criteria. Uncertainties were resolved by discussion, and the students filled knowledge gaps by using resources such as a textbook or the Internet. Even if an analytic approach was prioritized, real-life experiences from the clinic were also used during reasoning. The complexity of the clinical reality was thus introduced into the situation. According to our observations, the students alternated freely between the two approaches to reasoning and did not appear to make a distinction between them. These findings support previous suggestions that combining the two approaches to clinical reasoning is more beneficial compared with the use of only one style in educational settings, as they promote each other [6,8,23].

### Interactions With Other Resources

The setting in our study was based on VPs, using a structure that supported making a diagnosis and suggesting appropriate management of the patient before the real-life outcome of the respective patient was revealed. The students’ interactions were clearly driven by the VP design, and the specific cases were always the central focus. Nevertheless, the students used several resources other than the VP software. A textbook, lecture notes, and the Internet were utilized to gather information that provided evidence needed for further reasoning and consolidation of knowledge. The actions within the learning activity were therefore not exclusively directed by the VP software. It is reasonable to assume that large variations in interactions were influenced by the possibility of accessing various other resources, as well as by the design of the VP scenario [24].

### The Dyad Setting

Our observations suggest that the peer setting in dyads was pivotal for the elaborate reasoning observed, as it provided constructive resistance while processing the VPs. It is reasonable to assume that part of this reasoning could have occurred internally within an individual in a nonpeer setting; however, the reasoning would not have been explicitly voiced; it would have occurred in silence, and it would certainly not have been critiqued or evaluated by a peer. Experimental designs have found that group cognition differs from individual cognition and that the dyad setting is productive for abstractions [18]. Peer learning settings have also been shown to have benefits in terms of increased awareness of the learning process, stimulation of reflections during problem solving, and strengthened confidence [25-27]. In terms of group size in shared reasoning, the dyad setting worked very well in this VP arrangement. In a previous study comparing triads with individual settings using computer-based cases, no increased depth or elaborations were identified [28]. Nonetheless, the dyad constellation has been shown to be beneficial in clinical skills settings [29,30]. To our knowledge, no comparison has been conducted between dyad and triad peer settings in the context of VP-based learning. Such a comparison would shed more light on the influence of the size of the peer group on the reasoning process.

The constructive resistance that forms part of peer contribution may help to adjust topics to individuals’ level of knowledge and experience. Still, a concern that has been raised is the risk of knowledge imbalance within the dyad, or incompatible pairs [31,32]. In the two sessions of this study, we could see variations of previous knowledge within different fields. It is worth noting that instances when one student took the lead and suggested how the pair should continue during the reasoning process were apparent. In our data, negotiations and arguments for specific decisions were observed in both sessions. From a learning perspective, this might lead to consolidation of knowledge for the leading person while the more reflective peer contributes with different perspectives and suggestions. In session #2, one student was repeatedly referring to clinical experiences as a basis for the reasoning, whereas her peer was more analytical. In both sessions, different perspectives contributed to a richer picture, and disagreements were resolved during reasoning. During this process, the peer with a higher level of knowledge within a specific field had to provide arguments for their reasoning. However, one could assume that if one peer repeatedly displays lack of knowledge and needs to rely on the other, the reasoning process and the learning experience may be hampered. A study of online dyad settings reported inconclusive results of learning outcomes when pairs were asymmetric in terms of their level of knowledge [32]. More research on knowledge symmetry within the pairs is needed to identify optimal ways to match learners according to their level of knowledge and thus best support their learning.

The arranged VP situation allowed the pair to take the time to reason broadly, ponder over certain issues, and even experiment with physical examination procedures. This slowed-down, broader reflection is rare during a time-constrained real-life patient encounter. The VP setting provides a less stressful milieu for reasoning and reduces the perceived demands on having to appear knowledgeable in front of patients and educators [33]. Throughout the task, the students adopted the role of a treating physician, navigating themselves toward the right diagnosis, and making decisions about future management. However, the educational setting prevailed and the observed emotional engagement was not at the same level as when, for example, enacting acute patient scenarios in full-scale manikin-based medical simulations [34]. In addition to acting professionally, student roles were also adopted during the sessions. For example, the students referred to lecture notes, negotiated how one should approach the task, and referred to their own previous learning processes. The clear benefit of the VP case approach is a less formal and less demanding climate, allowing the students to expand their reasoning and reflection beyond what is possible in a full-scale simulation scenario or an authentic patient encounter, where decisions have to be made instantly.

### Methodological Considerations

A major strength of this study was the in-depth analysis of the sessions. However, the low number of sessions studied could be considered a limitation, as it limits the generalizability of our observations when applying them to other contexts. Thus, the identified themes are neither exhaustive nor expected to be replicated in all settings. Moreover, the characteristics of the specific cases and the VP interface may have influenced the reasoning process, and therefore the findings cannot be generalized to any VP dyad setting. The methods of observation provided not only an increased awareness of how students’ reasoning processes function while exploring a VP case but also an insight into how peer interactions may relate to clinical reasoning. However, it is worth noting that the students were aware of the fact that they were being filmed during the sessions, and a social desirability effect may have impacted their interactions.

### Implications

By actively taking part in verbal reasoning and highlighting the process as well as the outcome, the students increase the meta-awareness of their reasoning [35]. Meta-awareness is related to diagnostic outcomes [36]. Such an awareness can be created in collaborative VP sessions and followed up further in seminars, during which the students reflect both on the reasoning process and clinical content. Even greater focus on reasoning strategies could possibly be achieved by taking notes or, for example, visualizing reasoning steps in a mind map and then discussing them in a follow-up seminar. A visual representation in adjunct to a VP system has recently been developed by other researchers [37].

The number of VPs in this setting was small. Therefore, they do not substantially contribute to the nonanalytic aspect of expert reasoning. However, the dyad setting contributes to an in-depth encounter with a few cases supporting the analytic thinking. This analytic thinking process, and the awareness thereof, requires cognitive efforts and time to be processed. VP scenarios and the surrounding educational framework should therefore be designed carefully, taking the benefits of reasoning into consideration [38].

VP activities can be organized in different ways; these different arrangements may influence both the learning process and learning outcomes [24]. When integrating VPs, it is logistically tempting to instruct the students to work with the VP scenarios individually and independently on their own accord. This type of integration design has the clear benefit of flexibility but relies on the students’ own motivation and discipline. Edelbring et al found that fewer students (49% compared with 65%-96% of the students in a course) accessed the VPs, and these students also accessed fewer VP cases (an average of 1.27 out of 4 cases available) in an individual flexible integration compared with two different settings with scheduled follow-up seminars [39]. A link between VP course integration arrangements and student efforts to access and make use of VPs has been identified in various VP settings [40,41]. In this respect, arranged dyad settings may contribute to improved learning outcomes as a result of shared reasoning and provide a means for students to delve deeper and more broadly into the VP cases. Shared reasoning is clearly a means to better educational outcomes and fosters the collaborative and reflective practice needed in health care [13,19].

### Conclusions

The dyad setting enabled a stepwise investigation of VP cases and elaborated reasoning. Both analytic and nonanalytic reasoning occurred during the interactions. The VP activity also triggered interactions with other sources, which served as tools for information gathering and contributed to consolidation of knowledge. The VP design and the dyad arrangement enabled reasoning and rigorous learning processes that are unlikely to occur in individual settings.

## Appendix

Multimedia Appendix 1 Supplementary methods information.

## Abbreviations

NSAID: nonsteroidal anti-inflammatory drug
RA: rheumatoid arthritis
VP: virtual patient
