# Supplementary material for: Increasing Reasoning Awareness: Video Analysis of Students’ Two-Party Virtual Patient Interactions
Source: JMIR Med Educ. 2018 Feb 27;4(1):e4. doi: 10.2196/mededu.9137 (PMC5849799; doi:10.2196/mededu.9137)
Supplement: Multimedia Appendix 1 [file mededu_v4i1e4_app1.pdf]

Virtual patient descriptions

In session #1, the VP scenario was based on a 72-year-old man suffering from myalgia, morning stiffness, and headache. The investigation was intended to lead the students to diagnose polymyalgia rheumatica (PMR).

In session #2, the VP case was based on a 51-year-old man diagnosed with ankylosing spondylitis. His clinical presentation included stiffness and loss of function in the back, weight loss, and an elevated erythrocyte sedimentation rate. The students had to justify the right diagnosis; they had to rule out differential conditions and justify treatment strategies.
